# Taxonomic and distributional updates on the Arabian species of the genus *Palpita* Hübner, 1808 (Lepidoptera, Crambidae, Spilomelinae) 

**DOI:** 10.3897/BDJ.14.e199345

**Published:** 2026-07-27

**Authors:** Michael Seizmair, Alrabea Ishag, Hathal Mohammed Al Dhafer

**Affiliations:** 1 SNSB Bavarian State Collection of Zoology Munich, Munich, Germany (Independent Associated Scientist), Munich, Germany SNSB Bavarian State Collection of Zoology Munich, Munich, Germany (Independent Associated Scientist) Munich Germany; 2 King Saud University Museum of Arthropods, Department of Plant Protection, College of Food and Agriculture Sciences, King Saud University, Riyadh, Saudi Arabia King Saud University Museum of Arthropods, Department of Plant Protection, College of Food and Agriculture Sciences, King Saud University Riyadh Saudi Arabia; 3 King Saud University, College of Food and Agriculture Sciences, Riyadh, Saudi Arabia King Saud University, College of Food and Agriculture Sciences Riyadh Saudi Arabia

**Keywords:** Arabian Peninsula, morphology, new species, Pyraloidea, Saudi Arabia, taxonomy

## Abstract

**Background:**

The genus *Palpita* Huebner, 1808 presently comprises 166 valid species and is, thus, amongst the three most diverse genera in the subfamily Spilomelinae. The genus is represented in all zoogeographical main zones. In the Afrotropical zone, the genus is represented by a total of 21 species, 20 of which have been known exclusively from the Afrotropical zone to date. The Afrotropical zone reaches its northern boundaries in the southern parts of the Arabian Peninsula, which include southern Oman (province Dhofar), Yemen and the Sarawat Mountain chains in the south of Saudi Arabia. From the Arabian Peninsula, only two species are known to date, namely *Palpita vitrealis* (Rossi, 1794) and the recently described *Palpita subflavalis* Seizmair, 2022.

**New information:**

One further species new to science, *Palpita digitalis* **sp. nov**., is added to the fauna of the Arabian Peninsula, based on material from southern Saudi Arabia. The adults and the male genitalia are described and illustrated. The female genitalia are unknown. The new species is closest both in external and in internal morphology to the Aftrotropical *Palpita irroratalis* (Hampson, 1912) and *Palpita tsisabiensis* Maes, 2004 and to the Oriental *Palpita argoleuca* (Meyrick, 1938). The diagnostic characters in the male genitalia are listed. Taxonomic and distributional updates are given for *Palpita subflavalis* Seizmair, 2022, based on recent material from Saudi Arabia and Oman. External character states and the male genitalia are re-described. The species is reported as new to the fauna of Saudi Arabia. A determination key to the Arabian species of the genus *Palpita* Hübner, 1808 and to their closest Afrotropical and Oriental relatives is provided.

## Introduction

The genus *Palpita* Huebner, 1808 presently comprises 166 valid species and is, thus, amongst the three most diverse genera in the subfamily Spilomelinae (Nuss et al. 2025). It is represented in all zoo-geographical main zones. The genus has been attributed to the tribe Margaroniini (Mally et al. 2019). The genus is externally characterised by white ground, cellular spots and subterminal fasciae. Apomorphies of the genus in the internal morphology include the presence of saccular processes in the male genitalia, the presence of deciduous cornuti and the presence of paired signa in the female genitalia (Inoue 1996, Inoue 1997). In the Afrotropical zone, the genus is represented by a total of 21 species, 20 of which have to date been known exclusively from the Afrotropical zone (De Prins and De Prins 2025). The Afrotropical zone reaches its northern boundaries in the southern parts of the Arabian Peninsula, which include southern Oman (province Dhofar), Yemen and the Sarawat Mountain chains in the south of Saudi Arabia. Even though the fauna of the Arabian Peninsula is pre-dominated by Afrotropical elements, it is also influenced by the Palaearctic and Oriental zones, as has been shown for the Papilionoidea (Larsen 1984) and the Noctuoidea (Hacker 2016). From there, only two species of the genus *Palpita* Huebner, 1808 are known to date, namely *Palpita vitrealis* (Rossi, 1794) and the recently described *Palpita subflavalis* Seizmair, 2022. This paper presents further material of the genus from the southern Arabian Peninsula collected in the Asir mountain chain and Mount Fayfa in Saudi Arabia. One part of the sample is attributed to a further Arabian species, new for science, *Palpita digitalis* **sp. nov**. The other part is attributed to *Palpita subflavalis* Seizmair, 2022. The material significantly extends the presently known distribution range of the latter species to the southern Sarawat mountain chain in Saudi Arabia.

## Materials and methods

### Sampling

The material presented in this study is part of samples collected in Saudi Arabia in the Rayda Reserve (province Asir) in research projects of the KSMA in the years 2014 and 2015, in the Fayfa Mts (province Jizan) in the years 2022 – 2025, in the surroundings of Taif (province Mekka) in 2022 and in Oman (province Dhofar) in 2026. The specimens were captured at night by means of light traps, equipped with UV tubes and UV power-LEDs. The UV power-LEDS cover a wave spectrum of 365 nm – 385 nm (LepiLED, Nichia, Tokushima, Japan; EntoLED, Starlight, Weissenburg, Germany).

### Preparation, Dissection, Digital Image Processing

The adults were photographed after relaxation and subsequent preparation with a CANON EOS M6 Mark II under a MP-E-65 mm zoom or with the LEICA imaging system of the KSMA. Image stacking was done with Helicon Remote, Version 4.5.3. For examining the genitalia, slide-mounting techniques were applied on the specimens according to the protocol described by Robinson (1976). The genitalia were prepared with Motic (SMZ-171) and Meiji stereomicroscopes. The slides were photographed with a ToupCam c-mount camera (ToupTek Inc., Zhejiang, China). Image post-processing was performed by means of Adobe Photoshop PS, Version 26.11.0.

### Morphological Analyses and Comparisons

Analyses of wing pattern characters and morphological structures in the specimens were done on the images. Structural ratios were calculated on the images by means of the imaging software ToupView, Version 1.0 (ToupTek Inc., Zhejiang, China). Statistical measures (mean values, standard deviations) were calculated in MS Excel 2016, Version 5028. For the taxonomic assessment of the specimens, all known species both of the Afrotropical, Palaearctic and Oriental zones were checked. This meant reviewing both original descriptions and recent literature and comparing the specimens under investigation with images of type specimens and genitalia figures (Walker 1859, Walker 1866, Plötz 1880, Butler 1898, Hampson 1899, Hampson 1912, Hampson 1913, Hampson 1918a, Hampson 1918b, Meyrick 1933, Meyrick 1934, Meyrick 1937, Marion and Viette 1956, Maes 2004, Slamka 2013, Seizmair 2022, Alipanah and Slamka 2023, De Prins and De Prins 2025).

### Terminology and Abbreviations

The descriptions of external and internal character states follow the terminology in Inoue (1996) and Inoue (1997).

Abbreviations

CMS = Collection Michael Sezmair, Gröbenzell, Germany, KSMA = King Saud University Museum of Arthropods, n = size / cardinality of a sample, ± SD = Standard Deviation

## Taxon treatments

### Palpita subflavalis

Seizmair, 2022

6D079FB5-B5E2-53D8-B50F-99191483698A

Palpita subflavalis Seizmair, 2022. Seizmair (2022): 69-70. Type locality: Oman, Dhofar, Jebel al Qamar, Road 47, 20 km E Sarfait.

#### Materials

- **Type status:** Other material. **Occurrence:** catalogNumber: 25KSMA09; recordedBy: Al Dhafer, H.; Fadl, H., Abd Eldayem, M., El Torkey, A., El Gharbawy, A.; individualCount: 1; sex: male; lifeStage: adult; occurrenceID: 7D3E39DD-2C9A-57C8-A79D-D6FC37BE73F5; **Location:** country: Saudi Arabia; countryCode: SA; stateProvince: Asir; municipality: Abha; locality: Raydah Reserve; verbatimElevation: 2285m; verbatimLatitude: 18° 11.766; verbatimLongitude: 42° 24.315; **Event:** eventDate: 20-X-2014; **Material Entity:** disposition: KSMA
- **Type status:** Other material. **Occurrence:** catalogNumber: 26KSMA005; recordedBy: Al Dhafer, H.; Fadl, H., Abd Eldayem, M., El Torkey, A., El Gharbawy, A.; individualCount: 1; sex: male; lifeStage: adult; occurrenceID: 35DA3264-AC9C-5AA1-84A4-7A8D97BED4D0; **Location:** country: Saudi Arabia; countryCode: SA; stateProvince: Asir; municipality: Abha; locality: Raydah Reserve; verbatimElevation: 1851m; verbatimLatitude: 18° 11.679; verbatimLongitude: 42° 24.691; **Event:** eventDate: 05-III-2015; **Material Entity:** disposition: KSMA
- **Type status:** Other material. **Occurrence:** catalogNumber: 24KSMA008; recordedBy: Al Dhafer, H.; Fadl, H., Abd Eldayem, M., El Torkey, A., El Gharbawy, A.; individualCount: 1; sex: male; lifeStage: adult; occurrenceID: ABEA2DB5-7F29-5E14-ADE8-FFA138A04872; **Location:** country: Saudi Arabia; countryCode: SA; stateProvince: Asir; municipality: Abha; locality: Raydah Reserve; verbatimElevation: 1614m; verbatimLatitude: 18° 11.749; verbatimLongitude: 42° 23.345; **Event:** eventDate: 04-III-2015; **Material Entity:** disposition: KSMA
- **Type status:** Other material. **Occurrence:** catalogNumber: 24KSMA013; recordedBy: Al Dhafer, H.; Fadl, H., Abd Eldayem, M., El Torkey, A., El Gharbawy, A.; individualCount: 1; sex: female; lifeStage: adult; occurrenceID: D6F2C51A-2FB6-5F46-B007-291EE849D9DC; **Location:** country: Saudi Arabia; countryCode: SA; stateProvince: Asir; municipality: Abha; locality: Raydah Reserve; verbatimElevation: 2387m; verbatimLatitude: 18° 11.884; verbatimLongitude: 42° 24.435; **Event:** eventDate: 20-I-2015; **Material Entity:** disposition: KSMA
- **Type status:** Other material. **Occurrence:** catalogNumber: 24KSMA014; recordedBy: Al Dhafer, H.; Fadl, H., Abd Eldayem, M., El Torkey, A., El Gharbawy, A.; individualCount: 1; sex: male; lifeStage: adult; occurrenceID: 412BDF2C-D200-5118-BADC-B7D474D3E67E; **Location:** country: Saudi Arabia; countryCode: SA; stateProvince: Asir; municipality: Abha; locality: Raydah Reserve; verbatimElevation: 2820m; verbatimLatitude: 18° 12.265’; verbatimLongitude: 42° 24.744’; **Event:** eventDate: 07-VI-2014; **Material Entity:** disposition: KSMA
- **Type status:** Other material. **Occurrence:** catalogNumber: 26KSMA004; recordedBy: Al Dhafer, H.; Fadl, H., Abd Eldayem, M., El Torkey, A., El Gharbawy, A.; individualCount: 1; sex: female; lifeStage: adult; occurrenceID: 1AFBA118-D75B-5DAD-9A35-A074E8AA81F1; **Location:** country: Saudi Arabia; countryCode: SA; stateProvince: Asir; municipality: Abha; locality: Raydah Reserve; verbatimElevation: 2820m; verbatimLatitude: 18° 12.265’; verbatimLongitude: 42° 24.744’; **Event:** eventDate: 07-VI-2015; **Material Entity:** disposition: KSMA
- **Type status:** Other material. **Occurrence:** catalogNumber: 26KSMA002; recordedBy: Al Dhafer, H.; Fadl, H., Abd Eldayem, M., El Torkey, A., El Gharbawy, A; individualCount: 1; sex: female; lifeStage: adult; occurrenceID: 5810F6E2-AACB-5246-8EBD-37101369C850; **Location:** country: Saudi Arabia; countryCode: SA; stateProvince: Baha; municipality: Al Baha; locality: Shada Al Ala; verbatimElevation: 1563m; verbatimLatitude: 19° 50.329’; verbatimLongitude: 41° 18.604’; **Event:** eventDate: 27-I-2015; **Material Entity:** disposition: KSMA
- **Type status:** Other material. **Occurrence:** catalogNumber: 26KSMA006; recordedBy: Al Dhafer, H.; Fadl, H., Abd Eldayem, M., El Torkey, A., El Gharbawy, A; individualCount: 1; sex: male; lifeStage: adult; occurrenceID: 43AC8EAB-F016-5911-AEF3-B337007FE2A3; **Location:** country: Saudi Arabia; countryCode: SA; stateProvince: Baha; municipality: Al Baha; locality: Shada Al Ala; verbatimElevation: 1611m; verbatimLatitude: 19° 50.411’; verbatimLongitude: 41° 18.686’; **Event:** eventDate: 27-I-2015; **Material Entity:** disposition: KSMA
- **Type status:** Other material. **Occurrence:** catalogNumber: 22GP026; recordedBy: Seizmair, M.; individualCount: 3; sex: female; lifeStage: adult; otherCatalogNumbers: 22GP028, 22GP029; occurrenceID: E5024EA9-23F5-54A2-BD72-52E5F5A07512; **Location:** country: Saudi Arabia; countryCode: SA; stateProvince: Mekka; municipality: Taif; locality: Al Shafa; verbatimElevation: 1700m ; verbatimLatitude: 21° 3.722'; verbatimLongitude: 40°19.200'; **Event:** eventDate: 03-V-2022; **Material Entity:** disposition: CMS
- **Type status:** Other material. **Occurrence:** catalogNumber: 22GP027; recordedBy: Seizmair, M.; individualCount: 1; sex: female; lifeStage: adult; occurrenceID: 80086349-6A59-517F-B784-F39859208B72; **Location:** country: Saudi Arabia; countryCode: SA; stateProvince: Baha; municipality: Al Baha; locality: Balhurashi; verbatimElevation: 2030m; verbatimLatitude: 19°49.737’; verbatimLongitude: 41°33.421’; **Event:** eventDate: 02-V-2022; **Material Entity:** disposition: CMS
- **Type status:** Other material. **Occurrence:** catalogNumber: 22GP069; recordedBy: Seizmair, M.; individualCount: 1; sex: female; lifeStage: adult; occurrenceID: 47C6C05A-B760-5064-8544-D3E107231366; **Location:** country: Saudi Arabia; countryCode: SA; stateProvince: Jizan; municipality: Fayfa; locality: Ayban Belgazi; verbatimElevation: 630m; verbatimLatitude: 17°15.740'; verbatimLongitude: 43° 4.512'; **Event:** eventDate: 24-IX-2022; **Material Entity:** disposition: CMS
- **Type status:** Other material. **Occurrence:** catalogNumber: 25GP031; recordedBy: Seizmair, M.; individualCount: 1; sex: female; lifeStage: adult; occurrenceID: 91AE5AEC-AB3A-58AE-83B1-2463786359FE; **Location:** country: Saudi Arabia; countryCode: SA; stateProvince: Jizan; municipality: Fayfa; locality: Al Kashah; verbatimElevation: 830m; verbatimLatitude: 17°14,359'; verbatimLongitude: 43°41,2178'; **Event:** eventDate: 11-II-2024; **Material Entity:** disposition: CMS
- **Type status:** Other material. **Occurrence:** catalogNumber: 22GP071; recordedBy: Seizmair, M.; individualCount: 2; sex: female; lifeStage: adult; otherCatalogNumbers: 22GP072; occurrenceID: 0E2F7647-418A-52B8-9DE1-76FD785FD40A; **Location:** country: Saudi Arabia; countryCode: SA; stateProvince: Jizan; municipality: Al Reeth; locality: Wadi Lajab, Khatwat Al Ain; verbatimElevation: 1300m; verbatimLatitude: 17°35.290'; verbatimLongitude: 42°55.950'; **Event:** eventDate: 27-IX-2022; **Material Entity:** disposition: CMS
- **Type status:** Other material. **Occurrence:** catalogNumber: 26GP004; recordedBy: Seizmair, M.; individualCount: 3; sex: male; lifeStage: adult; otherCatalogNumbers: 26GP005, 26GP006; occurrenceID: 37BB2327-B6E0-51B7-9CA9-AA916CE49B24; **Location:** country: Oman; countryCode: OM; stateProvince: Dhofar; municipality: Dhalkut; locality: Dhalkut; verbatimElevation: 150m; verbatimLatitude: 16°42.272'; verbatimLongitude: 53°14.144'; **Event:** eventDate: 23-I-2026; **Material Entity:** disposition: CMS

#### Description

**External characters (Figs 1a, b, 2a)**: Wingspan: 20.1–25.0 mm. Fore-wing length: 10.5–13.0 mm. Head: Antenna white in the flagellum, ciliae greyish-brown. Vertex and frons grey-scaled. Labial palpus porrect, dorsal and lateral scaling darkish-brown to ochreous sporadically interspersed with darkish-grey scales, ventrally with elongated greyish-white scales in segments 1 and 2, total length relative to the diameter of the eye 1.3±SD 0.09 (n = 9), ratio maximum length / maximum width 2.65 ± SD 0.18 (n = 9). Maxillary palpus porrect, brownish, very short, length relative to the length of the labial palpus 0.13 ± SD 0.02 (n = 4). Thorax: Dorsum, venter and tegula grey-scaled. Legs dorsally uniformly grey-scaled, tibia ventrally interspersed with brownish scales, tibial spurs significantly differing in length, with the longer spur three times as long as the shorter one. Fore- and hind-wing ground greyish-white irrorated with brownish scales, fore-wing with a yellowish-brown subcostal stripe ranging from the basis to the apex. Hind-wing terminal line marked as a series of yellowish-brown interneural spots. Fore- and hind-wing fringe greyish-white. Wings bare from any further maculation. Fore-wing triangular-shaped, elongate, 2.8 times as long as wide, apex pointed, slightly down-turned, tornus flattened anal border straight. Hind-wing 1.8 times as long as wide, apex rounded, tornus edged. Abdomen: Venter and dorsum greyish-white, presence of a greyish-white anal tuft.

**Male genitalia (Fig. 3a, b, c)**: Uncus with the neck constant in width, 4.7 ± SD0.99 (n = 5) times longer than wide, apex membranous, subtriangular to cordate in shape, laterally chaetose. Tuba analis membranous, length relative to the length of the uncus 1.3±SD 0.09 (n = 5), subscaphium absent. Valva 1.7 ± SD 0.07 times longer than wide, costal border strongly convex, slightly sclerotised, constant in width, narrow, ventral border concave, apical portion protuberant, elongated, constricted, chaetose. Sacculus with a dorso-medial invagination, posterior half tapered, presence of one distal saccular process, which is bifid, with the arms forming a right angle and significantly differing in length. The longer of the two arms is slender, constant in width, slightly curved towards the apex and 1.47 ± SD 0.07 (n = 5) times longer than the shorter one. The shorter of the two arms is of sub-triangular shape. The juxta is of trapezoid shape and projected antero- and postero-laterally. The saccus is rounded, u-shaped, with a posteriad-directed ventro-apical process. Phallus with the posterior portion strongly widened, vesical sclerotisation marked as three deciduous cornuti, one of them very short, half as long as the longer ones, where the two longer cornuti are equal in length. Of the two longer cornuti, the posteriormost cornutus is basally curved, acuminate and spiculose, the anteriormost cornutus is straight and medially dilated.

**Figure 5.**
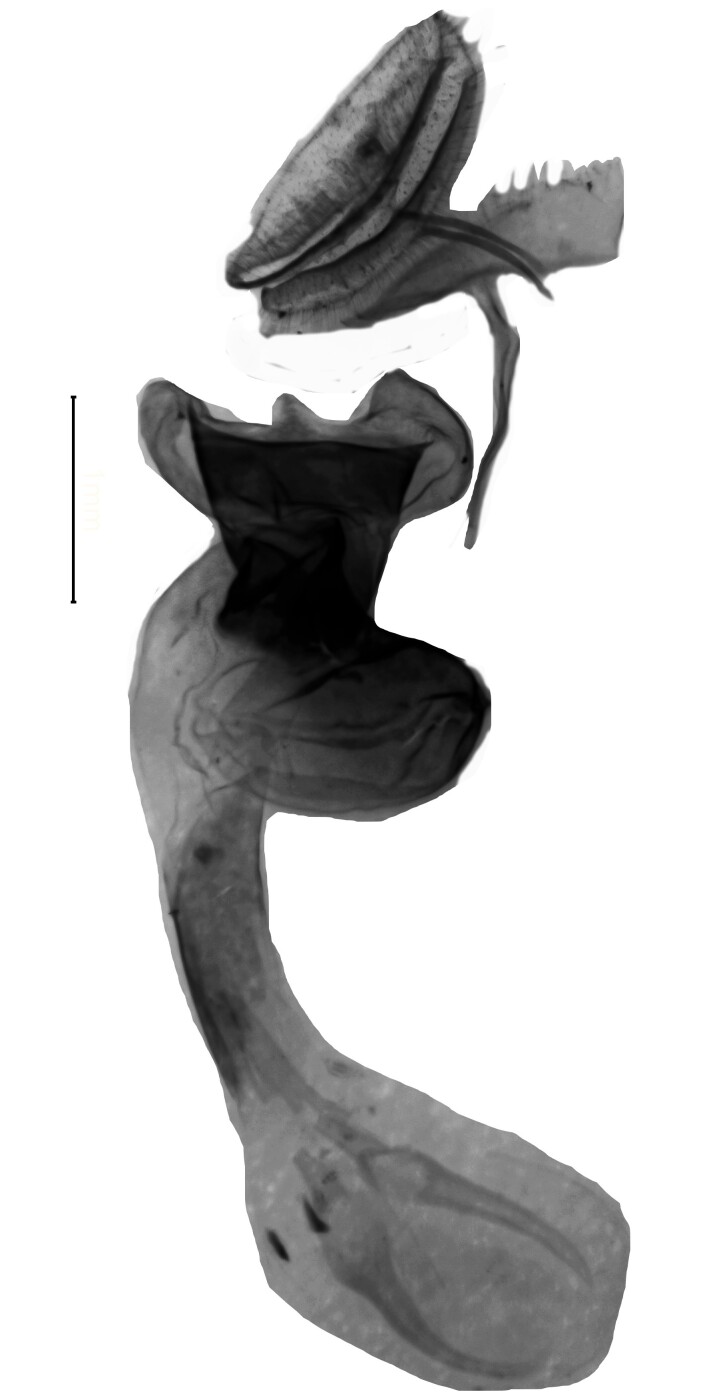
Female genitalia of *Palpita subflavalis* Seizmair, 2022, slide no. 22GP071: Scale bar = 1.0 mm.

**Female genitalia (Fig. 5)** Papilla analis ovate, 3.5 times longer than wide, chaetae very short. Posterior apophyses slender, unprojected, constant in width, slightly bent. Anterior apophyses projected in the posterior fifth, 1.5 times longer than the anterior apophyses. Antrum membranous strongly sclerotised, of sub-quadrangular shape. Ductus bursae with the posterior half strongly dilated, cordate and asymmetrical with extensive strongly sclerotised areas, 1.43 times wider than long (± SD 0.05, n = 6) and the anterior half weakly sclerotised, elongate, 3.37 times longer than wide (± SD 0.40, n = 6). Corpus bursae ovate, twice as long as wide (±SD 0.20, n = 5), equal in length with the ductus bursae (± SD 0.10, n = 5), signa very small to tiny, slender, acuminate, thorn-shaped.

#### Diagnosis

The diagnostic character states in the male genitalia known from the original description are the number and shape of the saccular processes – one distal bifid saccular process, the sclerotisation of the vesica marked as three cornuti, one short, slender the other two ones deciduous and acuminate. By these character states, the species is clearly differentiated from the externally close congeners of the Oriental *Palpita vitrealis* (Rossi, 1794) species group as listed in the original diagnosis (Seizmair 2022) from the Afrotropical *Palpita irroratalis* (Hampson, 1912) and *Palpita tsisabienis* Maes, 2004. Further diagnostic character states, which have become known by the analysis of the present material are the presence of dorso-medial invagination in the sacculus and the trapezoid shape of the laterally projected juxta. Furthermore, the species is differentiated in the female genitalia from the *P. vitrealis* species group by the presence of tiny, thorn-shaped signa, in the strongly dilated and strongly sclerotised posterior ductus bursae and by the strongly sclerotised, trapezoid-shaped antrum.

#### Distribution

Oman - province Dhofar. New to Saudi Arabia - southern Saravat mountains (Fayfa Mts., Asir Mts., southern Hejaz Mts.).

#### Notes

The re-description revises the attribution of character states in the original description, namely in external characters of the head profile and in characters in the male and female genitalia (sacculus, costa, signa). The head profile is re-figured (Fig. 2) at an enhanced resolution level compared with the figure in the original description. The male genitalia show variation in the size of the valva, in the length of the arms of the distal sacculus. The female genitalia exhibit variation in the size of the dilated posterior ductus bursae and in the length of the anterior ductus bursae. There are no intersexual differences, neither in wing maculation nor in wing shape.The data do not allow attribution of the variability to geographic separation between the samples from Dhofar and Saudi Arabia. The authors thus refrain from drawing taxonomic consequences at subspecific level.

### Palpita digitalis

Seizmair, Ishag, Al-Dhafer sp. nov.

273639BE-4681-5572-B300-71C2E9E3BF3F

DAD72139-E65F-4186-876F-14CD67AE53F0

#### Materials

- **Type status:** Holotype. **Occurrence:** catalogNumber: 25KSMA011; recordedBy: Al Dhafer, H.; Fadl, H., Abd Eldayem, M., El Torkey, A., El Gharbawy, A.; individualCount: 1; sex: male; lifeStage: adult; occurrenceID: 0C5A3446-A81F-52C8-8C44-6BD8DEEFA828; **Location:** country: Saudi Arabia; countryCode: SA; stateProvince: Asir; municipality: Abha; locality: Raydah Reserve; verbatimElevation: 2387 m; verbatimLatitude: 18° 11.884'; verbatimLongitude: 42° 24.435'; **Event:** eventDate: 26-IV-2014; **Material Entity:** disposition: KSMA
- **Type status:** Paratype. **Occurrence:** catalogNumber: 24KSMA011; recordedBy: Al Dhafer, H.; Fadl, H., Abd Eldayem, M., El Torkey, A., El Gharbawy, A.; individualCount: 1; sex: male; lifeStage: adult; occurrenceID: 6965653B-D712-5E32-8B07-57B323C0344D; **Location:** country: Saudi Arabia; countryCode: SA; stateProvince: Asir; municipality: Abha; locality: Raydah Reserve; verbatimElevation: 2387 m; verbatimLatitude: 18° 11.884'; verbatimLongitude: 42° 24.435'; **Event:** eventDate: 26-IV-2014; **Material Entity:** disposition: KSMA

#### Description

**External characters (Figs 1c, d, 2b)**: Wingspan: 25.0 mm. Fore-wing length: 12.3 mm. Head: Antenna greyish-white in the shaft, flagellum and ciliae. Frons and vertex with tufts of long greyish-white scales, vertex laterally with a row of darkish-brown scales along the compound eye. Labial palpus equal in length with the diameter of the eye, twice as long as wide, porrect in segments 1 and 2, obliquely down-turned in segment 3, with short yellowish-brown scales, ventrally with a narrow and elongate tuft of white scales covering segments 1 and 2. Maxillary palpus obliquely upturned, short, truncate, length relative to the length of the labial palpus 20%, yellowish-brown, with the tip of segment 4 white-scaled. Thorax: Dorsum, venter and tegula greyish-white scaled. Fore-wing twice as long as wide. Apex pointed, down-turned. Termen convex. Tornus edged. Ground white, sporadically interspersed with blackish pigments. Costal stripe yellowish-brown terminating shortly below the apex. Terminal line blackish, reduced to a series of interneural spots. Fringe greyish-white. Hind-wing 1.2 times longer than wide. Apex and tornus rounded. Ground, terminal line and fringe as for the fore-wing. There is no further maculation in the fore- nor in the hind-wing. The undersides are identical to the upper sides. Legs dorsally greyish-white scaled, ventrally yellowish-brown. Abdomen: Venter and dorsum greyish-white scaled.

**Male genitalia (Fig. 4)**: Uncus composed of a neck and a beak-shaped apex. The neck is slender, basally slightly widened, medially bent. The transition to the beak-shaped apex is smooth, with the apex dorsally covered with very short chaetae. Tuba analis elongate, of sub-triangular shape, basally with a neck-shaped constriction, length relative to the length of the uncus 1.3. Scaphium and sub-scaphium absent. Valva 1.4 times longer than wide, medially strongly widened. Costal border strongly convex, constant in width. Sub-apical membranous portion with fine, short chaetae, broad, rather short. Apex medially rounded. Ventral border straight. Sacculus basally dilated and rounded, post-basally narrowed, with one dorso-distal process. The dorso-distal process is basally widened, forming a right-angled structure with the rest of the sacculus, with its length relative to the maximum width of the valva 50%. Juxta shield-shaped, acuminate posteriorly, elongate, twice as long as wide, reaching up to the inferior ridge of the transtilla. Transtilla strongly widened, of rhomboid shape, with a short, well-developed transtillum inferior. Vinculum 1.2 times wider than long, saccus v-shaped, ventral sclerotisation marked as an elongate, weakly sclerotised strip. Phallus strongly dilated in its posterior portion, vesical sclerotisation marked as two slightly bent digitiform cornuti, differing in length by a factor of two and a further small, straight, acuminate cornutus, the lengths of the three cornuti differing by a factor of two.

**Female genitalia**: The female genitalia are unknown.

#### Diagnosis

*Palpita digitalis* sp. nov. is closest to the Afrotropical *P. irroratalis* and *P. tsisabiensis* and to the Oriental *P. argoleuca* externally and in the male genitalia. Externally, the three species share the complete bareness from any black maculation in the fore- and hind-wing. In the male genitalia, they share the shape of the uncus, composed of a neck and a beak-shaped apex and the number and shape of saccular processes – one simple dorso-distal process, which forms a right-angled structure with the rest of the sacculus (Inoue 1997, Maes 2004). The diagnostic characters are listed in Table 1. The comparison is based on the adult images, male genitalia figures and diagnoses in Inoue (1997) and Maes 2004. It should be noted that that the two comparative species *P. irroratalis* and *P. tsisabiensis* are very close in the male genitalia, in which they differ exclusively in the length of the uncus, in the width of the saccus and in the length of the ventro-apical sclerotisation in the valva (Maes 2004). One further species sharing the wing maculation with the new species and its closest relatives is *Palpita candicantis* Inoue, 1997, which is, however, distinguished from the new species and each of its comparative species by the presence of two widely-separated distal saccular processes (Inoue 1997).

#### Etymology

The epitheton refers to one of the differential character states in the male genitalia, namely the digitiform cornuti in the vesica.

#### Distribution

At present, only known from the type material from south-western Saudi Arabia (prov. Asir).

#### Ecology

The type material was exclusively collected in the Junipera zone of the Afromontane escarpment in the Asir Mts.

#### Biology

The life history (premature stages, food plant selection) is unknown and subject to further research.

## Identification Keys

### Key to the presently known Arabian species of *Palpita* and their Afrotropical and Oriental relatives

**Table:** 

1	Presence of two widely-separated distal saccular processes	*P. candicantis*
–	Presence of one distal saccular process	2
2	Distal saccular process bifid, apex of the uncus cordate to sub-triangular-shaped	*P. subflavalis*
–	Distal saccular process simple, forming a right angle with the rest of the sacculus, apex of the uncus beak-shaped	3
3	Saccular process constant in width, with a dorso-lateral appendix, juxta posteriorly invaginated	4
–	Saccular process posteriorly narrowed, bare from appendix, juxta bare from invagination	*P. digitalis***sp.nov**.
4	Dorso-lateral appendix of the saccular process basally broad, triangular-shaped, fibula present in the centre of the valva	*P. argoleuca*
–	Dorso-lateral appendix of the saccular process spatulate, fibula absent in the centre of the valva	5
5	Uncus and ventro-apical sclerotisation in the valva elongate, saccus broadly triangular	*P. tsisabiensis*
–	Uncus and ventro-apical sclerotisation in the valva shortened, saccus narrowed	*P. irroratalis*

## Discussion

One new species of the genus *Palpita* 1808 was described from the southern Arabian Peninsula, namely *Palpita digitalis* sp. nov. *Palpita subflavalis* Seizmair, 2022 was reported as new to the fauna of Saudi Arabia. The species has been known exclusively from the southern part of the Arabian Peninsula to date. The taxon was re-described, based on further material from Saudi Arabia and Oman. A determination key to the Arabian species of the genus and to their Afrotropical relatives was provided. The life history, premature stages, host plant selection and abundance of the Arabian species *P. digitalis* sp. nov. and *P. subflavalis* are subject to further research.

## Supplementary Material

XML Treatment for Palpita subflavalis

XML Treatment for Palpita digitalis

## Figures and Tables

**Figure 1a.**
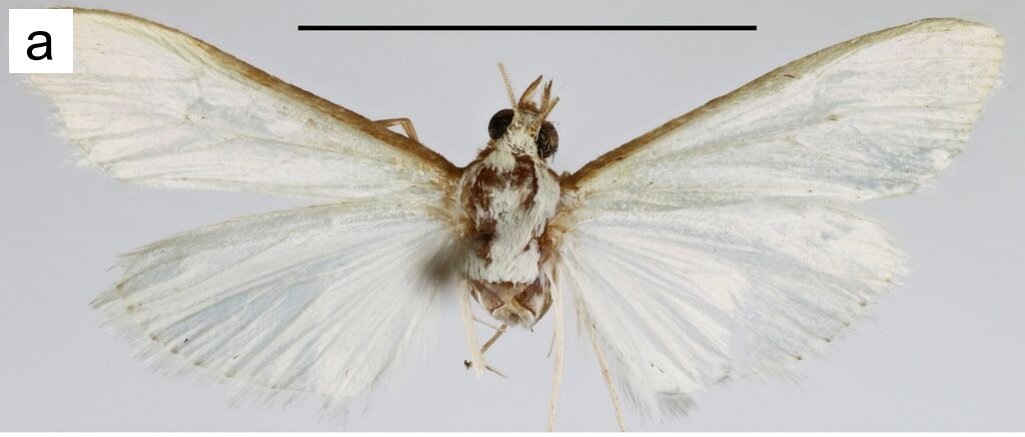
*Palpita subflavalis* Seizmair, 2022, male, slide no. 25KSMA009;

**Figure 1b.**
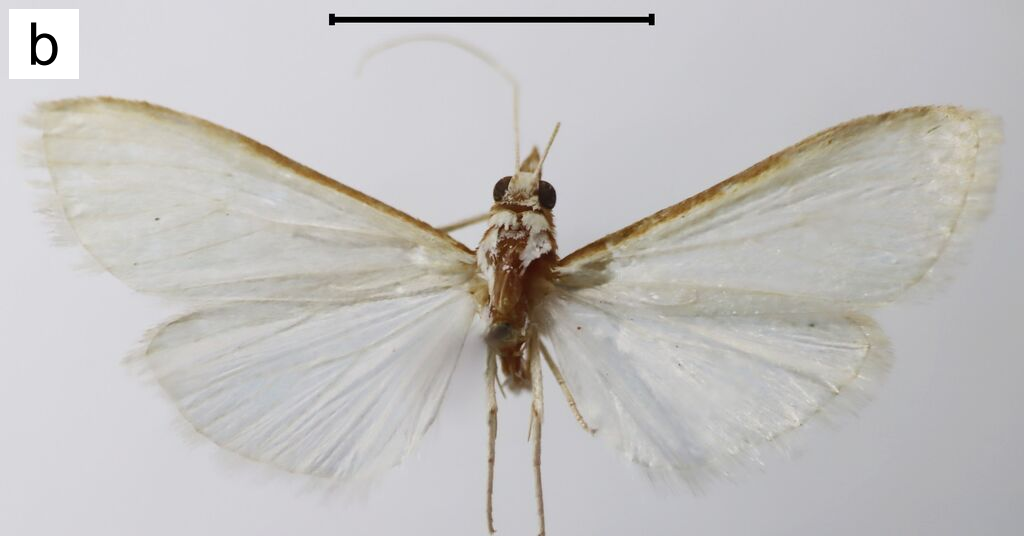
*Palpita subflavalis* Seizmair, 2022, female, slide no. 22GP028;

**Figure 1c.**
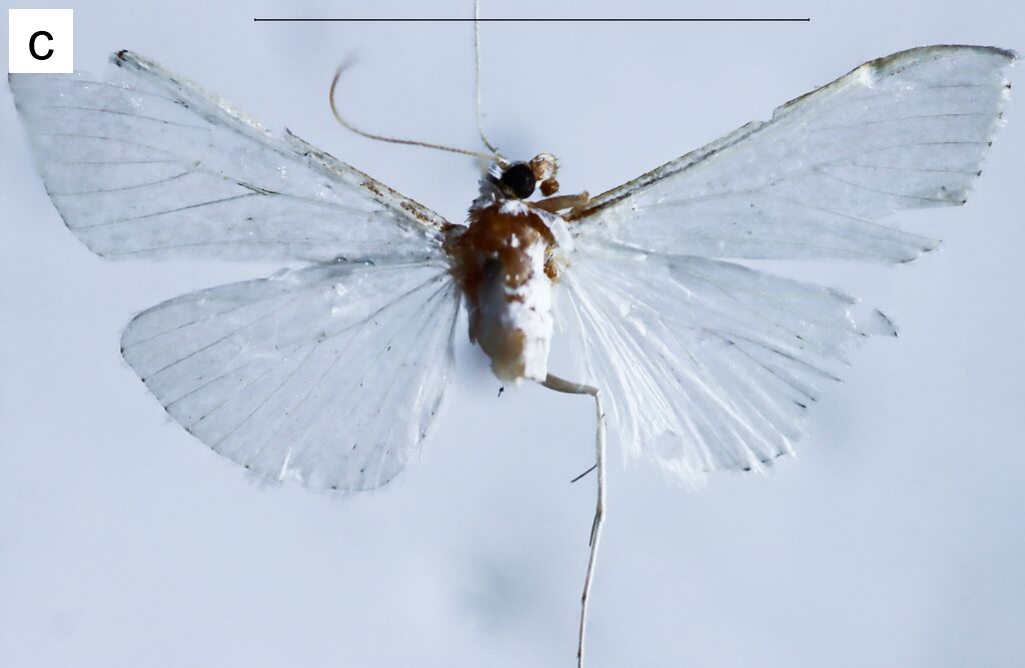
*Palpita digitalis*, sp.nov., holotype, male, slide no. 25KSMA011;

**Figure 1d.**
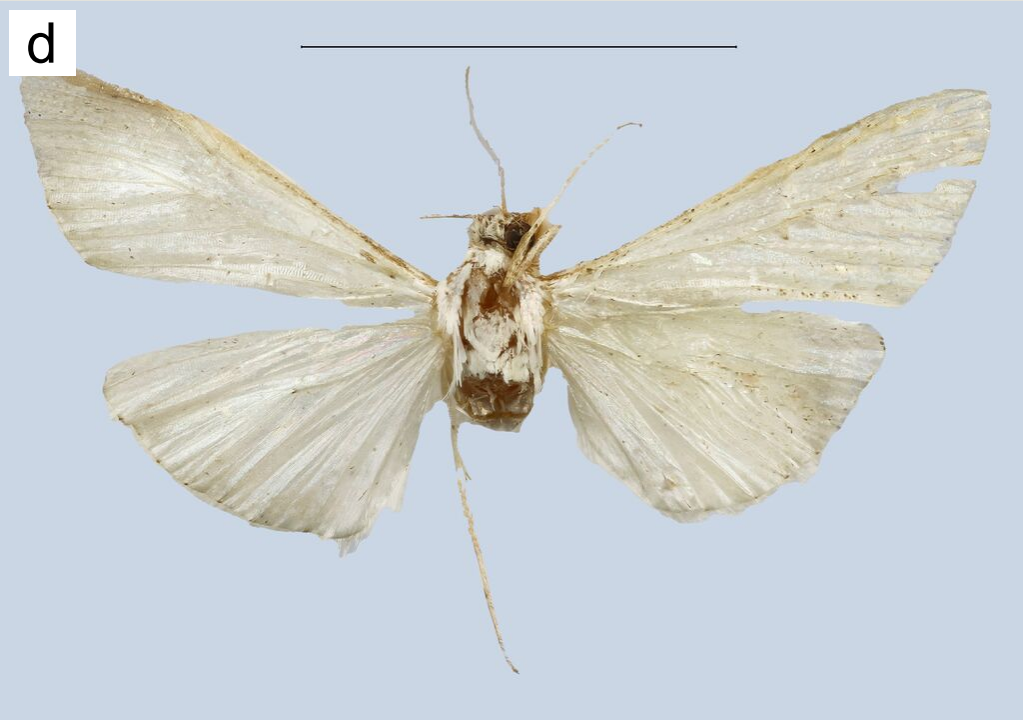
*Palpita digitalis*, sp.nov., paratype, male, slide no. 24KSMA011.

**Figure 2a.**
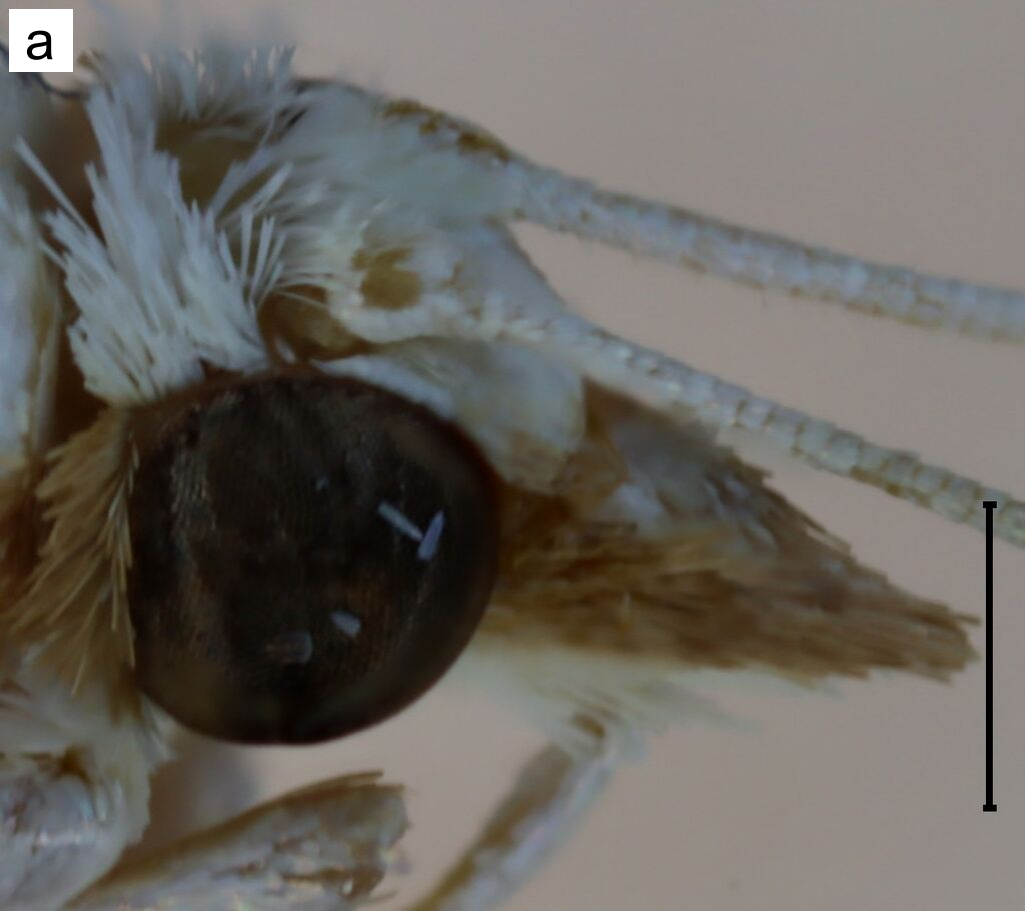
*Palpita subflavalis* Seizmair, 2022, female, slide no. 22GP028;

**Figure 2b.**
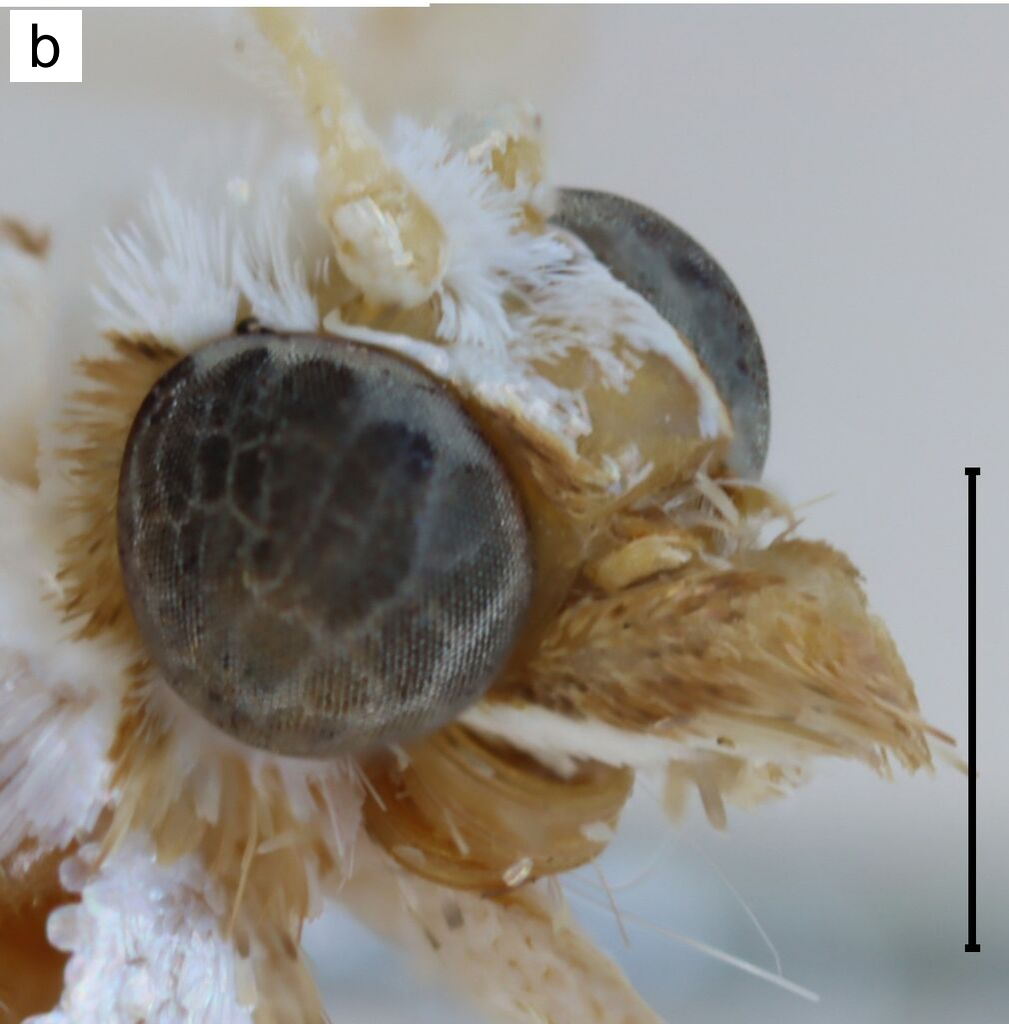
*Palpita digitalis* sp. nov., male holotype, slide no. 25KSMA011.

**Figure 3a.**
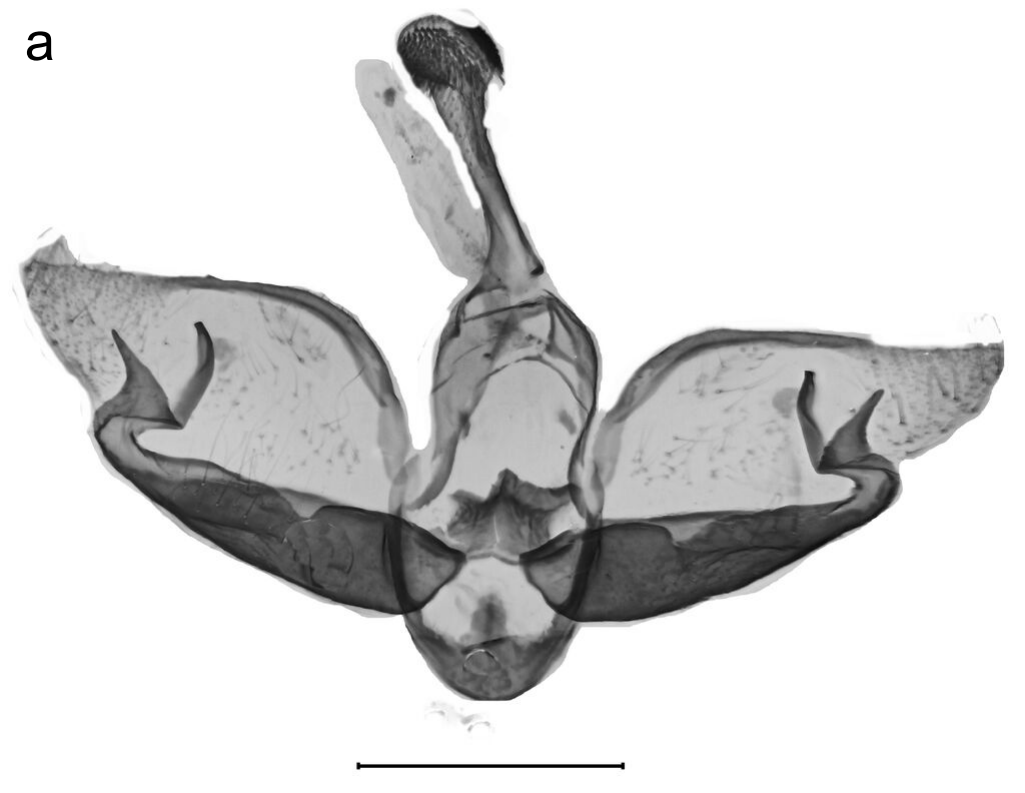
capsule, phallus omitted (slide no. 26KSMA006);

**Figure 3b.**
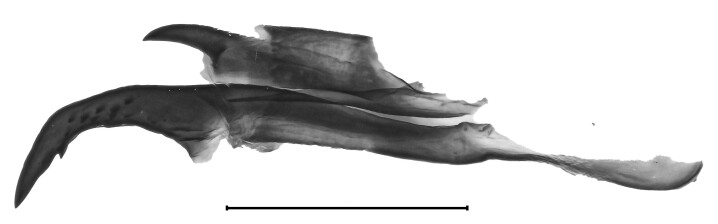
phallus (slide no. 26KSMA006);

**Figure 3c.**
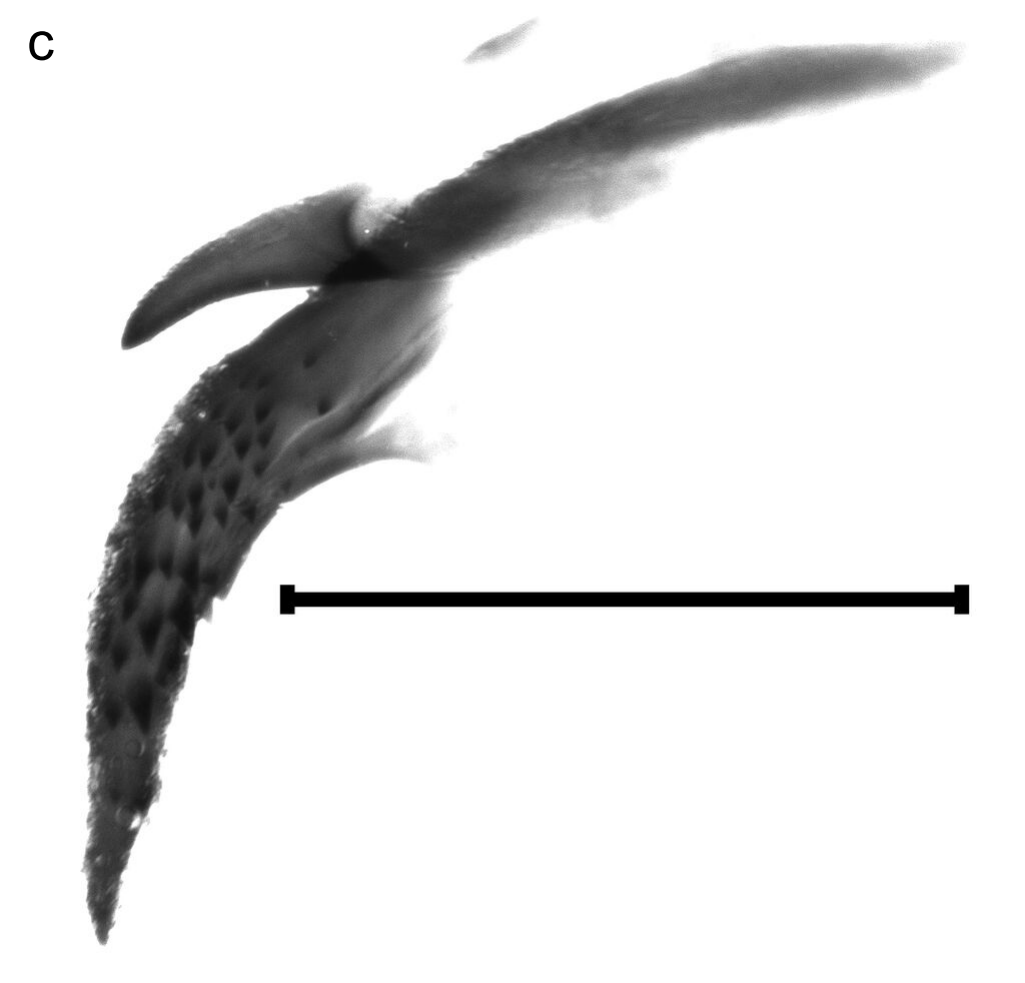
cornuti, close-up (slide no 25KSMA008).

**Figure 4a.**
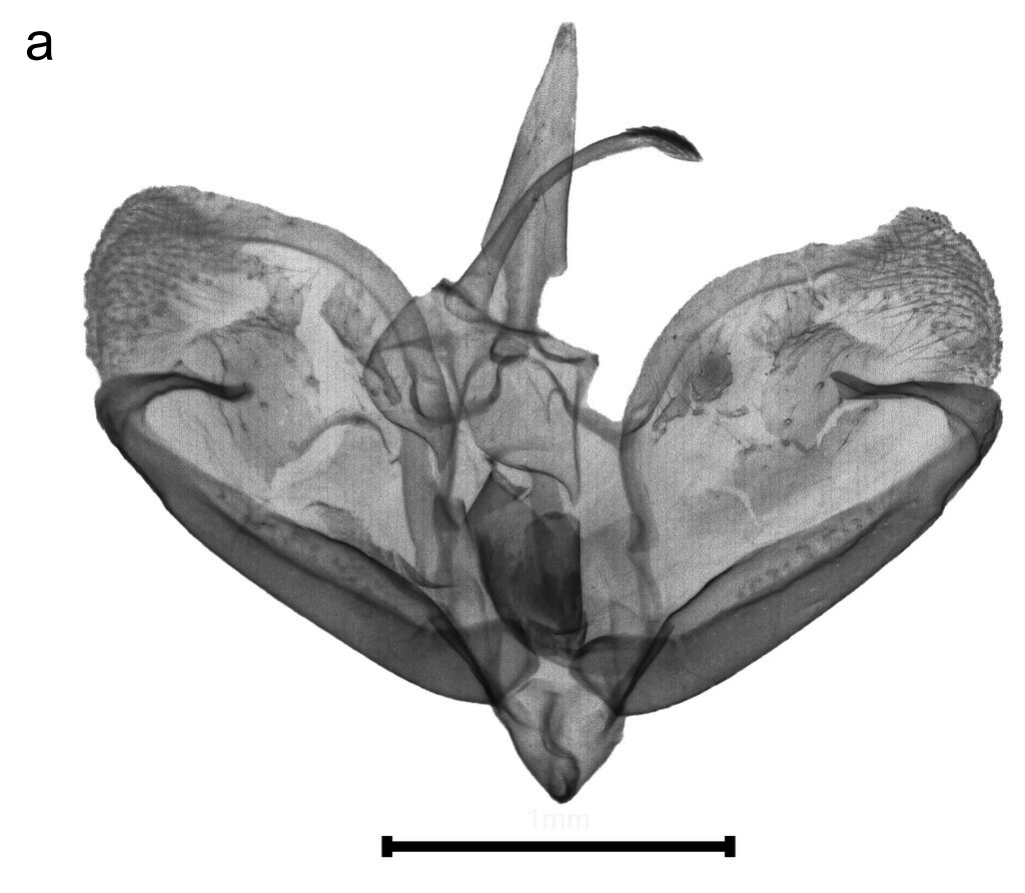
Genitalia capsule;

**Figure 4b.**
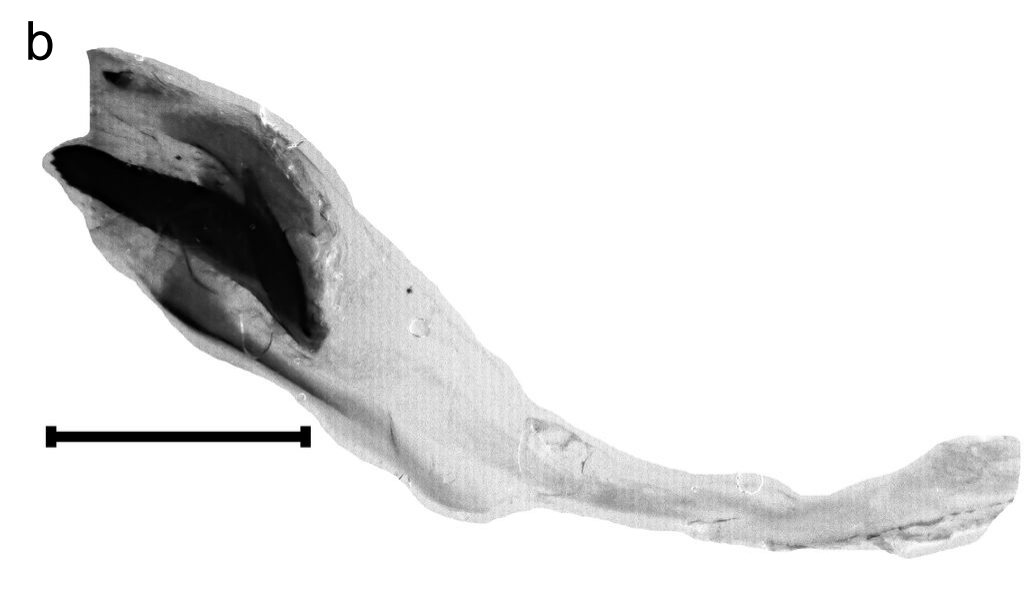
phallus apodeme;

**Figure 4c.**
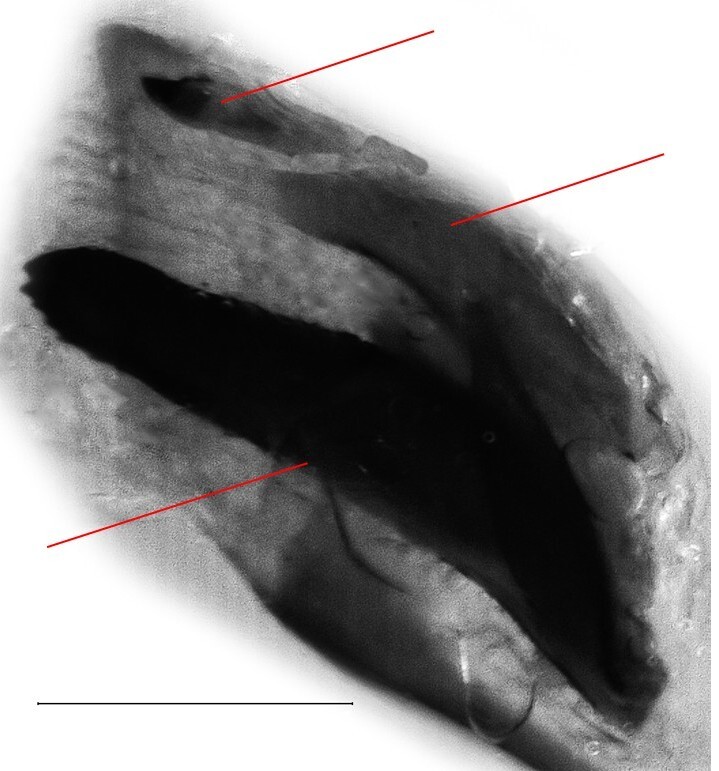
Close-up, cornuti.

**Table 1.** Diagnosis - Differential characters in the male genitalia distinguishing *P. digitalis* **sp.nov**. from its closest relatives *P. tsisabiensis*, *P. irroratalis* and *P. argoleuca*.

	*P. digitalis***sp.nov**.	*P. tsisabiensis*	*P. irroratalis*	*P. argoleuca*
Shape of the juxta – presence of posterior invagination (0 = absent, 1 = present)	0	1	1	1
Shape of the juxta – relation width / length (0 = ≈ 0.5, narrowed, elongated, 1 = ≈ 1.4, strongly widened)	0	1	1	0
Shape of the valva – relation width / length (0 = ≈ 0.60, narrowed, 1 = ≈ 0.75, widened)	1	0	0	0
Presence of a fibula (process) in the valva	0	0	0	1
Shape of the vinculum (0 = narrowed, 1 = broadly triangular)	0	1	0	0
Shape of the uncus – prominence of the beak, width relative to the width of the posterior end of the neck (0 = not prominent, ≈ 1.0, 1 = prominent, ≈ 1.6)	0	1	1	0
Shape of the distal saccular process (0 = posteriorly strongly narrowed, bare from appendix, 1 = constant in width, with a sclerotised dorso-lateral appendix, 2 = constant in width, with a basally strongly, widened, triangular-shaped dorso-lateral process)	0	1	1	2
Shape of the cornuti (0 = digitiform, rounded, 1 = broadened, triangular-shaped, 2 = elongate, slender, acuminate)	0	1	1	2
Number of cornuti	3	2	2	1
